# Nephrostomy tube versus double J ureteral stent in patients with malignant ureteric obstruction. A systematic review and meta-analysis of comparative studies

**DOI:** 10.1590/S1677-5538.IBJU.2022.0225

**Published:** 2022-05-31

**Authors:** Vineet Gauhar, Giacomo Maria Pirola, Simone Scarcella, Maria Vittoria De Angelis, Carlo Giulioni, Emanuele Rubilotta, Marilena Gubbiotti, Ee Jean Lim, Yu Xi Terence Law, Marcelo Langer Wroclawski, Ho Yee Tiong, Daniele Castellani

**Affiliations:** 1 Ng Teng Fong General Hospital Department of Urology Singapore Singapore Department of Urology, Ng Teng Fong General Hospital, Singapore, Singapore; 2 San Donato Hospital Department of Urology Arezzo Italy Department of Urology, San Donato Hospital, Arezzo, Italy; 3 Le Marche Polytechnic University Faculty of Medicine School of Urology Ancona Italy Faculty of Medicine, School of Urology, Le Marche Polytechnic University, Ancona, Italy; 4 University of Verona Azienda Ospedaliero Universitaria of Verona Department of Urology Verona Italy Department of Urology, Azienda Ospedaliero Universitaria of Verona, University of Verona, Verona, Italy; 5 Singapore General Hospital Department of Urology Singapore Singapore Department of Urology, Singapore General Hospital, Singapore, Singapore; 6 National University Hospital Department of Urology Singapore Singapore Department of Urology, National University Hospital, Singapore, Singapore; 7 Hospital Israelita Albert Einstein São Paulo SP Brasil Hospital Israelita Albert Einstein, São Paulo, SP, Brasil; 8 Faculdade de Medicina do ABC Santo André SP Brasil Faculdade de Medicina do ABC - FMABC, Santo André, SP, Brasil; 9 BP – A Beneficência Portuguesa de São Paulo São Paulo SP Brasil BP – A Beneficência Portuguesa de São Paulo, São Paulo, SP, Brasil; 10 University Surgical Cluster National University Hospital Department of Urology Singapore Singapore Department of Urology, University Surgical Cluster, National University Hospital, Singapore, Singapore; 11 Azienda Ospedaliero Universitaria Ospedali Riuniti di Ancona Urology Unit Ancona Italy Urology Unit, Azienda Ospedaliero Universitaria Ospedali Riuniti di Ancona, Ancona, Italy

**Keywords:** Ureteral Obstruction, Nephrostomy, Percutaneous, Urinary Diversion

## Abstract

**Purpose:**

We aimed to perform a systematic review to assess perioperative outcomes, complications, and survival in studies comparing ureteral stent and percutaneous nephrostomy in malignant ureteral obstruction.

**Materials and Methods:**

This review was performed according to the Preferred Reporting Items for Systematic Reviews and Meta-Analyses framework. Meta-analyses were performed on procedural data; outcomes; complications (device-related, accidental dislodgement, febrile episodes, unplanned device replacement), dislodgment, and overall survival. Continuous variables were pooled using the inverse variance of the mean difference (MD) with a fixed effect, and 95% confidence interval (CI). The incidences of complications were pooled using the Cochran-Mantel-Haenszel method with the random effect model and reported as Odds Ratio (OR), and 95% CI. Statistical significance was set two-tail p-value <0.05

**Results:**

Ten studies were included. Procedure time (MD −10.26 minutes 95%CI −12.40-8.02, p<0.00001), hospital stay (MD −1.30 days 95%CI −1.69 − −0.92, p<0.0001), number of accidental tube dislodgments (OR 0.25 95% CI 0.13 – 0.48, p<0.0001) were significantly lower in the stent group. No difference was found in mean fluoroscopy time, decrease in creatinine level post procedure, overall number of complications, interval time between the change of tubes, number of febrile episodes after diversion, unplanned device substitution, and overall survival.

**Conclusion:**

Our meta-analysis favors stents as the preferred choice as these are easier to maintain and ureteral stent placement should be recommended whenever feasible. If the malignant obstruction precludes a stent placement, then PCN is a safe alternative.

## INTRODUCTION

Malignant ureteral obstruction is the consequence of secondary, extrinsic compression/infiltration of the ureter causing obstruction in different cancers. Mechanisms include intraluminal ureteral tumour invasion, ureteral entrapment or compression by retroperitoneal/pelvic lymphadenopathy or metastasis, and as a consequence of retroperitoneal fibrosis induced by surgery, chemotherapy, and radiotherapy (1).

The primary reason for referral to urologists is to evaluate the most appropriate type of urinary diversion and its feasibility accounting for both disease and patient characteristics.

Urologists aim to relieve urinary obstruction, reduce symptoms, and improve renal function while preserving patient quality of life and potentially prolonging overall survival as many of these patients have ongoing treatments (2). Urinary diversion can be achieved externally via a percutaneous nephrostomy tube or internally using a double J ureteral stent (3).

The endoscopic approach may be technically difficult and at times even impossible in advanced pelvic or retroperitoneal disease with high failure rates particularly in pelvic malignancies. Indeed, the success of retrograde ureteral stenting in patients with pelvic malignancy is usually significantly lower in patients with extrinsic ureteral obstruction compared with those with internal ureteral obstruction due to non-progression of the hydrophilic guide and non-identification of the ureteral meatus (4). On the other hand, the percutaneous approach may negatively affect patient quality of life being more invasive and often associated with a greater incidence of infection, bleeding, discomfort, and accidental tube displacement (5). Frail patients may be even more reluctant in accepting long indwelling nephrostomy tubes that need regular change with further worsening of quality of life (2). The choice must be balanced according to operator experience and patient's choice whilst evaluating the patient clinical condition and life expectancy. Unlike temporary urinary diversion in acute urinary obstruction, there are currently no guidelines or consensus for the optimal approach in malignant ureteral obstruction.

The present study aimed to systematically review the literature to assess perioperative outcomes, complications, and survival in studies comparing double J ureteral stent and percutaneous nephrostomy in malignant ureteral obstruction to help clinicians in taking an informed decision on urinary diversion choices by understanding the nuances of both interventions.

## EVIDENCE ACQUISITION

### Literature search

We aimed to perform a meta-analysis comparing outcomes in patients with ureteral obstruction secondary to malignancies. This systematic review was performed according to the 2020 Preferred Reporting Items for Systematic Reviews and Meta-Analyses (PRISMA) framework. A comprehensive literature search was performed on 6th December 2021, using MEDLINE, EMBASE, and Cochrane Central Controlled Register of Trials (CENTRAL). The following term and Boolean operators were used: (ureteral stent OR urinary diversion OR double-J) AND (nephrostomy tube OR external urinary drainage) AND (extrinsic ureteral obstruction OR ureteral obstruction OR cancer ureteral obstruction). No date limits were imposed. The search was restricted to English papers, searching comparative studies between the two urinary diversions. Animal and paediatric studies were excluded. Additional articles were sought from the reference lists of the included articles. The review protocol was registered in PROSPERO (CRD42022297668).

### Selection criteria

The PICOS (Patient Intervention Comparison Outcome Study type) model was used to frame and answer the clinical question. P: patients with external ureteral obstruction due to malignancy; Intervention: ureteral stent; Comparison: nephrostomy tube; Outcome: procedural time, fluoroscopy time, post-procedural complications, hospital stay, decrease in creatinine, episodes of accidental dislodgment, and overall survival; Study type: prospective randomized studies, retrospective, or prospective non-randomized studies.

### Study Screening and Selection

Two independent authors screened all retrieved records through Rayyan Intelligent Systematic Review (https://www.rayyan.ai/). Discrepancies were solved by a third author. Studies were included based on PICOS eligibility criteria. Meeting abstracts, case reports, reviews, letters to editor, and editorials were excluded. The full text of the screened papers was selected if found relevant to the present review. The screening was further expanded by performing a manual search based on the references of the full-text relevant papers.

### Data Synthesis and Statistical Analysis

Outcomes were split into three main domains: i) procedural data (operative time, fluoroscopy time, and the number of device replacements); ii) outcomes (postoperative creatinine, hospital stay, and overall survival); iii) complications (device-related, accidental dislodgement, febrile episodes, and unplanned device replacement).

Continuous variables (procedural time, fluoroscopy time, length of stay, creatinine, overall survival) were pooled using the inverse variance of the mean difference (MD) with a random effect, 95% confidence interval (CI), and p-values. The incidences of complications and accidental tube dislodgment were pooled using the Cochran-Mantel-Haenszel method with a random effect model and reported as Odds Ratio (OR), 95% CI, and p-values. Statistical significance was set two-tail p-value <0.05. Study heterogeneity was assessed utilizing the I^2^ value. Substantial heterogeneity was defined as an I^2^ value between 75% and 100%. Significance was set at p-value <0.05 (two tails) and 95%CI. Meta-analysis was performed using Review Manager (RevMan) 5.4 software by Cochrane Collaboration. The quality assessment of the included studies was performed using the ROBINS-I for non-randomized studies (6).

## EVIDENCE SYNTHESIS

### Literature screening

**Figure 1:**
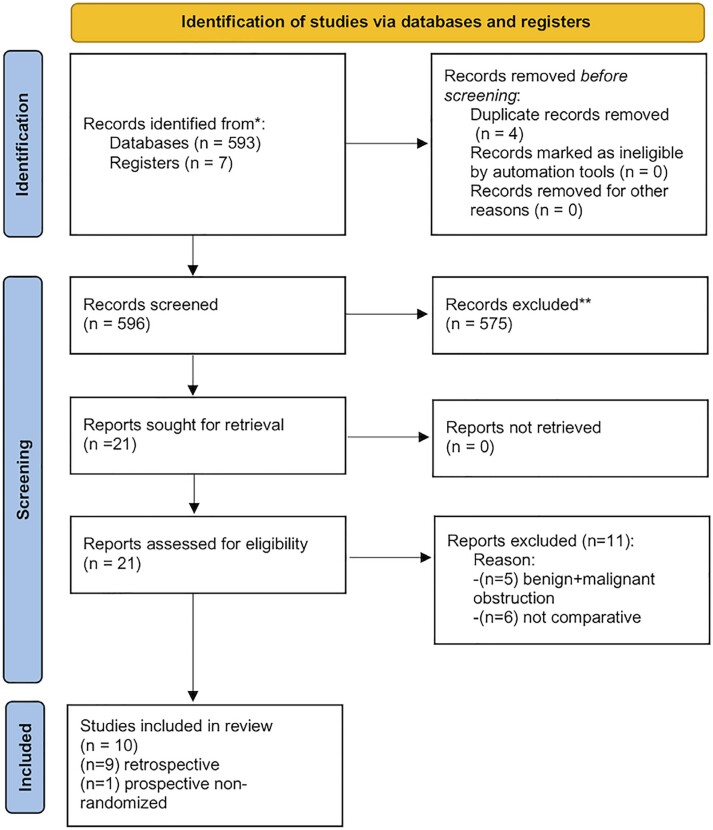
PRISMA flow diagram of the study.

Literature search retrieved 596 papers. After title and abstract screening, 575 records were excluded because they were not related to the study purpose. The full texts of the remaining 21 studies were assessed for eligibility. Eleven studies were further excluded due to missing data. Finally, ten studies were accepted and included (7–16). Among these, only one was prospective (7) while the others were retrospective studies (8–16). No randomized study was found. Figure-1 shows the 2020 PRISMA flow diagram. Study characteristics are summarized in Table-1.

**Table 1:** Characteristics of the included studies.

Authors	Type of study: 1 RCT; 2 retrospective; 3 prospective	Ureteral stent patients (n)	Nephrostomy patients (n)	Total (n)	Type of cancer (absolute number) in ureteral stent	Type of cancer (absolute number) in nephrostomy tube
De Lorenzis et al. 2020 (8)	Retrospective	27	24	51	Upper Gastrointestinal tract (n=5); Lower Gastrointestinal tract (n=22)	Upper Gastrointestinal tract (n=4); Lower Gastrointestinal tract (n=20)
Gasparini et al. 1991 (9)	Retrospective	7	15	22	Ovarian cancer (n=2); cervical cancer (n=2); gastric cancer (n=1); colon cancer (n=1); prostate cancer (n=1)	Prostate cancer (n=1); Cervical cancer (n=3); Gastrointestinal (n=3); Ovarian cancer (n=1); Lymphoma (n=2); Unknown tumor (n=1); Bladder cancer (n=4)
Kanou et al. 2007 (10)	Retrospective	51	24	75	Cervix cancer (n=7); Rectal cancer (n=4); Prostate cancer (n=7); Bladder cancer (n=3); Ovarian cancer (n=2); Retroperitoneum tumor (n=1)	Lymphoma (n=2)
Ku et al. 2004 (11)	Retrospective	68	80	148	Not available	Not available
McCullough et al. 2008 (12)	Retrospective	31	26	57	Prostate cancer (n=5); bladder (n=5); colon (n=4); gynecological (n=7); breast (n=3); lymphoma (n=2); lung (n=1); others (n=4)	Prostate cancer (n=15); Bladder (n=7); Colon (n=3); gynecological (n=1)
Monsky et al. 2013 (7)	Prospective non randomized	15	15	30	Bladder (n=4); cervical (n=6); prostate (n=1); ovarian (n=2); endometrial (n=1); fallopian tube (n=1)	Bladder (n=5); cervical (n=3); uterine (n=2); prostate (n=2); colon (n=1); lymphoma (n=1); sarcoma (n=1)
Song et al. 2012 (13)	Retrospective	50	25	75	Cervical cancer (n=26); Endometrial cancer (n=22); Ovarian cancer (n=20); Uterine leiomyosarcoma (n=4); Vaginal carcinoma (n=1); Choriocarcinoma (n=2)	
Tan et al. 2019 (14)	Retrospective	69	20	89	Cervical cancer	Cervical cancer
Tibana et al. 2019 (15)	Retrospective	26	15	41	Bladder cancer (n=7); Uterine cancer (n=6); Metastatic colorectal cancer (n=4); Adenocarcinoma of the prostate (n=3); Sarcoma of the prostate (n=3); Colorectal adenocarcinoma (n=1); Retroperitoneal neuroendocrine tumor (n=1)	Uterine cancer (n=5); Bladder cancer (n=5); Prostate adenocarcinoma (n=3); Metastatic colorectal cancer (n=1); Ovarian cancer (n=1)
Zadra et al. 1987 (16)	Retrospective	27	53	80	Not available	Not available

### Study quality assessment

**Supplementary Figure 1:**
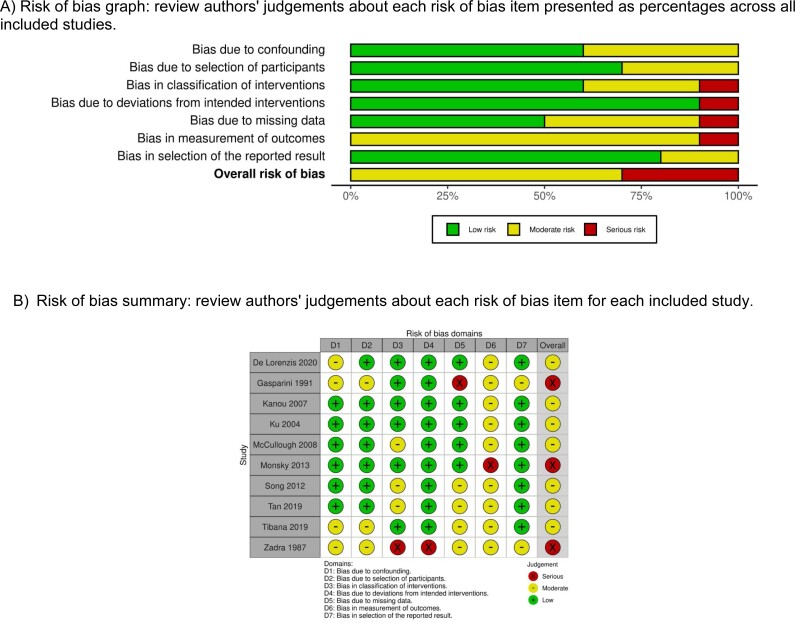
Risk of bias in non-randomized controlled trials (ROBINS-I)

Supplementary Figure-1 demonstrates the details of the quality assessment. Seven studies exhibited a moderate risk of bias for all quality criteria, while three showed a serious risk of bias. The most common risk factor for quality assessment was the risk of bias in the classification of interventions, bias due to deviations from intended interventions, bias due to missing data, and bias in the measurement of outcomes as the studies were retrospective in design.

### Procedural data

**Figure 2:**
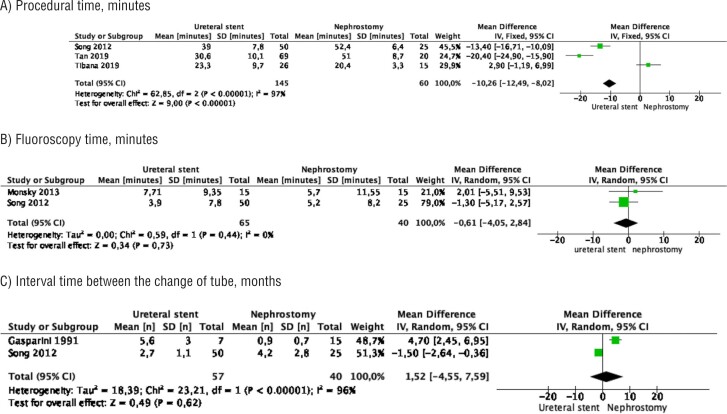
Meta-analysis of procedural data. A) procedure time; B) fluoroscopy time; C) interval time between the change of drainage tubes over time

Meta-analysis from 3 studies (145 cases in stent and 60 cases in nephrostomy) showed that the mean procedure time was significantly shorter in the stent group (MD −10.26 minutes, 95% CI −12.40 −8.02, p<0.00001). Study heterogeneity was substantial (I^2^ 97%) (Figure-2A).

Meta-analysis from 2 studies (65 cases in stent and 40 cases in nephrostomy) showed no difference between the two groups in mean fluoroscopy time (MD −0.61 minutes, 95% CI −4.05 – 2.84, p=0.73). Study heterogeneity was not significant (I^2^ 0%) (Figure-2B).

Meta-analysis from 2 studies (65 cases in stent and 40 cases in nephrostomy) showed no difference between the two groups in the mean interval time between the change of drainage tubes over time (MD 1.52 months, 95% CI −4.55 – 7.59, p=0.62). Study heterogeneity was substantial (I^2^ 96%) (Figure-2C).

### Outcomes

**Figure 3:**
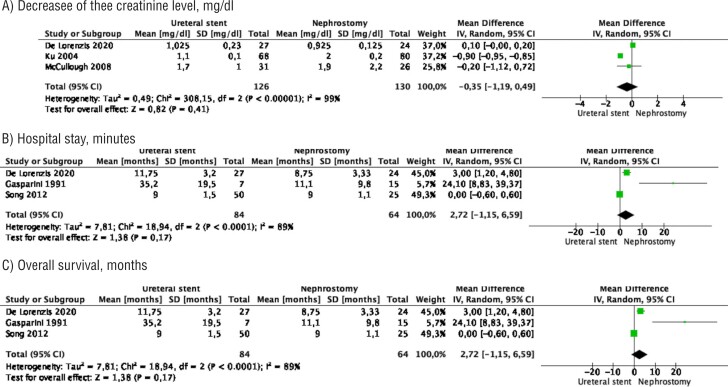
Meta-analysis of outcomes. A) decrease of the creatinine level after the procedure; B) hospital stay; C) overall survival.

Meta-analysis from 3 studies (126 cases in stent and 130 cases in nephrostomy) showed no difference between the two groups in the decrease of the creatinine level after the procedure (MD −0.35 mg/dL, 95% CI −1.19 − 0.49, p=0.41). Study heterogeneity was substantial (I^2^ 99%) (Figure-3A).

Meta-analysis from 3 studies (146 cases in stent and 69 cases in nephrostomy) showed a significantly shorter hospital stay in the stent group compared to the nephrostomy tube group (MD −1.30 day, 95% CI −1.69 − −0. 92, p<0.0001). Study heterogeneity was substantial (I^2^ 70%) (Figure-3B).

Meta-analysis from 3 studies (84 cases in stent and 64 cases in nephrostomy) showed no difference in the mean overall survival between the two groups (MD 2.72 months 95% CI −1.15 − 6.59, p=0.17). Study heterogeneity was substantial (I^2^ 89%) (Figure-3C).

### Complications

**Figure 4:**
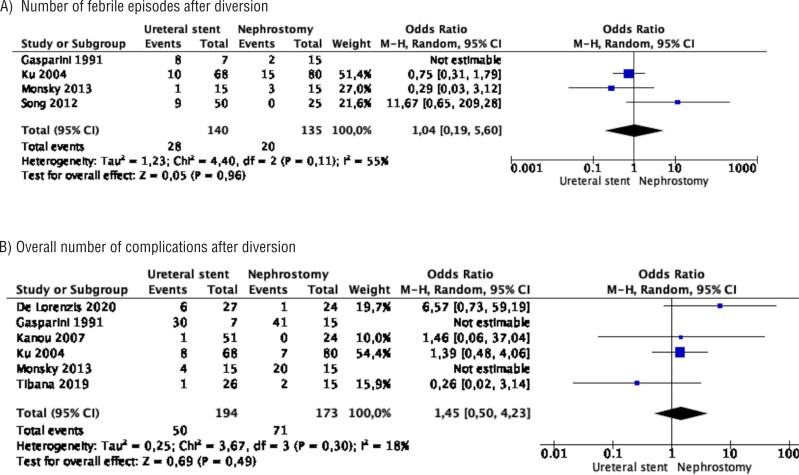
Meta-analysis of complications. A) number of febrile episodes after diversion; B) overall number of complications after diversion.

Meta-analysis from 4 studies (140 cases in stent and 135 cases in nephrostomy) showed no difference in the number of febrile episodes after diversion between the two groups (OR 1.04 95% CI 0.19 − 5.60, p=0.96). Study heterogeneity was moderate (I^2^ 55%) (Figure-4A).

Meta-analysis from 4 studies (140 cases in stent and 135 cases in nephrostomy) showed no difference in the overall number of complications after diversion between the two groups (OR 1.46 95% CI 0.72 − 2.95, p=0.30). There was no study heterogeneity (I^2^ 0%) (Figure-4B).

**Figure 5:**
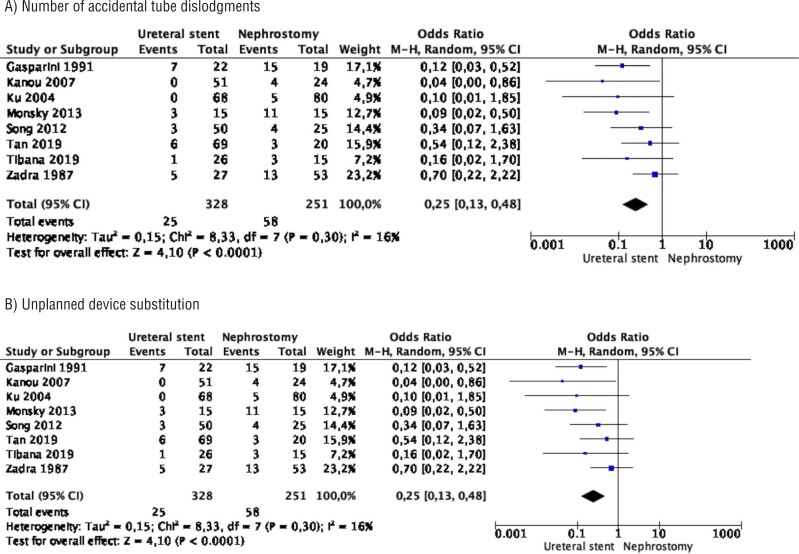
Meta-analysis of complications. A) number of accidental tube dislodgments; B) unplanned device substitution.

Meta-analysis from 8 studies (328 cases in stent and 251 cases in nephrostomy) showed that the number of accidental tube dislodgments was significantly lower in the stent group compared to the nephrostomy tube group (OR 0.25 95% CI 0.13 – 0.48, p<0.0001). Study heterogeneity was not important (I^2^ 16%) (Figure-5A).

Meta-analysis from 3 studies (134 cases in stent and 119 cases in nephrostomy) showed no difference in unplanned device substitution between the two groups (OR 0.41 95% CI 0.06 – 2.98, p=0.38). Study heterogeneity was moderate (I^2^ 43%) (Figure-5B).

## DISCUSSION

Malignant ureteral obstruction commonly affects patients with advanced cancers. Development of ureteral obstruction is slow and insidious, typically causing dull pain, associated with fatigue and lethargy. Malignant ureteral obstruction is often an ominous sign frequently associated with poor survival (17).

Patients are usually referred when clinical or radiological evaluation resulting from urinary stasis often with worsening kidney function is observed as a consequence of ureteral obstruction. Occasionally, acute symptoms may occur such as fever due to urinary infection or renal colic with nausea and vomiting due to a sudden increase in pressure or stretch of the ureteral lumen with the hypercontractility of the ureteral smooth muscle and subsequent activation of nociceptors of renal afferent nerves fibers by prostaglandins (18). Sometimes, *de novo* acute obstructive uropathy can be the first presenting sign of advanced pelvic cancers (19).

The management of patients with ureteral obstruction needs a multi-disciplinary approach involving urologists, oncologists, palliative care physicians, interventional radiologists, along with patients and caregivers. In most cases, obstruction is primarily asymmetrical. In the case of bilateral involvement, it is common practice to drain only the symptomatic kidney or the kidney with better function in asymptomatic patients. Although there are recommendations within cancer-specific guidelines, there is a lack of consensus as well as a strong piece of evidence to support the decision process on which modality of decompression has a better outcome (20). Often, at this late stage of malignancy, the quality of life is poor and therefore the ethics of palliative decompression have often been questioned (19). Patients with advanced malignancy are poor surgical candidates, and the option of no intervention should also be discussed since the procedures themselves are not without potential morbidity (3). Indeed, the mean survival has been reported to be 120-140 days even with decompression (19, 21). Considerable variability in survival time has been reported in the literature and it is therefore important to identify objective criteria that can be used to estimate a patient's prognosis. Lapitan et al. followed up a cohort of patients who had a malignant ureteral obstruction and assessed the outcomes of those who were decompressed and those who were not (22). The authors found that the 6-month survival of patients who underwent diversion was 38% compared with 28% of those who did not. By 12 months, both groups had the same survival of 16%. In our analysis, we found no difference in mean survival between the two groups, pointing out that the type of urinary diversion does not impact the overall survival.

Since considerable variability in survival time has been reported in the literature, it is therefore important to identify objective criteria that can be used to what type of diversion will probably minimize the impact on patients’ quality of life.

Our study showed that stent placement had a shorter operative time and hospital stay but mean change interval trend over time did not differ. The overall complication rate was also not different between the two approaches, but the accidental displacement was significantly higher in the nephrostomy group. We also found no differences in creatinine level decrease after decompression or complication rates between the two procedures. Therefore, the ureteral stent placement had better procedural results, similar efficacy, and fewer handling issues than percutaneous nephrostomy tube placement. Indeed, the endoscopic approach, which represents a less invasive procedure, ensured a faster discharge of patients and a lower risk of tube displacement. This last point is very relevant because ureteral stent placement avoided repeated and unnecessary treatments, which can be very troublesome in frail patients. For all these reasons, physicians should be inclined to treat patients with malignant ureteral obstruction first with a ureteral stent, whenever possible. However, the decision to choose either should rely more on identifying risk factors associated with disease progression and resource availability at the place of practice.

The prognostic stratification model by Lienert et al. and Ishioka et al. have identified some risk factors to help decision-making for percutaneous nephrostomy placement with a 3-month to one-year benefit seen only in low or favourable risk and intermediate-risk group (19, 21). In addition, survival has been demonstrated to differ among cancers causing ureteral obstruction. For instance, tumours that originate outside the true pelvis (such as breast, pancreatic and gastric cancers) have a worse prognosis whereas patients with prostate and gynaecological cancers have longer survival times (2). For patients predicted to have relatively long survival, conversion to an internal ureteral stent may be recommended for a better quality of life [6]. In our meta-analysis, ureteral stenting had a significant advantage over the percutaneous nephrostomy cohort in terms of fewer device dislodgements (OR 0.25 95% CI 0.13 – 0.48, p<0.0001), an important consideration in patients needing a longer duration of diversion, especially in the modern era where metallic stents are available and superior to conventional polymeric stents and can stay up to 1 year before the next change (23).

As per our meta-analysis, the stent group showed better procedural results, similar efficacy, and fewer handling issues than the percutaneous nephrostomy group, however, it is not uncommon to face ureteral stent insertion failure, particularly in those cases with cystoscopy evidence of bladder or ureteral invasion (2, 4). Bladder cancer and prostate cancer-causing ureteral obstruction have shown a far higher failure rate than that caused by colon or breast cancer, probably due to the former directly invading the trigone, causing both intrinsic and extrinsic obstruction, making stent insertion, not possible (24). We also found that there was no difference in unplanned intervention for a device substitution either due to a mechanical device malfunction or for clinical reasons such as increasing febrile episodes secondary to the implants. These have a significant bearing on quality of life and hence it may be easiest for a patient to manage a ureteral stent than a percutaneous nephrostomy tube. Moreover, our meta-analysis significantly favoured ureteral stent insertion as this minimized the hospital stay (MD −1.30 day 95% CI −1.69 − −0. 92, p<0.0001) with a similar procedure-related fluoroscopy time for both procedures. These factors can influence decision-making for clinicians when faced with malignant ureteral obstruction especially as these patients are a critically vulnerable cohort. In addition, the procedural cost can also be taken into account. Only one study reported data on cost analysis and showed that the average cost of stenting was significantly lower than percutaneous nephrostomy (US$164.10 vs. US$552.20, respectively) (15).

Specific to malignant ureteral obstruction related to genitourinary malignancies, Shekarriz et al. analysed 103 patients with advanced malignancies treated with palliative urinary diversion (stent or percutaneous nephrostomy) and found that prostate cancer patients had the longest median survival, although the difference did not reach significance (25). Instead, gynaecological cancer patients survived approximately 4-fold longer than those with bladder cancer. Among patients with bladder cancer, those presenting *de novo* with ureteral obstruction survived significantly longer than those in whom obstruction developed after diagnosis and treatment already administered (26). These are important considerations as often in these patients if there is any difficulty in cystoscopic access for stent placement a percutaneous nephrostomy should be the best consideration for immediate diversion in advanced malignancies (11). However, a concern for percutaneous nephrostomy is that tube may need to be changed more frequently due to blockage with a reported incidence of 0.4-37% in various studies and can lead to more febrile episodes due to infection with a reported incidence of 2-8% in various studies (27). This can make clinicians and patients reluctant for this intervention, but our meta-analysis showed no significant difference between the two groups in the mean interval time between change of drainage tubes or the number of febrile episodes.

Our study pointed out two important take-home messages. First, ureteral stenting represents a less invasive procedure and has more appeal for patients, ensuring a faster discharge of patients, a longer exchange interval, and a lower risk of tube displacement. This last point is very relevant because ureteral stent placement avoided repeated and unnecessary interventions, which can save costs and precious time for other palliative procedures. Second, if stent placement is technically not possible, since no differences were noted in complication rates and unplanned need for device substitution, patients can be safely advised that percutaneous nephrostomy is not an inferior choice as a primary drainage procedure, especially in the presence of a pelvic malignancy or in patients with shorter survival that requires only palliative relief of ureteral obstruction.

The present review has some limitations. The study is based only on retrospective studies and one prospective study with no randomized data and the number of patients in each study is relatively small. We argue that this reflects two reasons. First of all, a randomized study may not be feasible, because the choice of kidney decompression is mostly patient-tailored. Second, the few comparative studies could also reflect the low interest of the scientific community in this field that, conversely, deserves attention as a pivotal role in the palliative management of end-life cancer patients. We were not able to assess the quality of life after urinary diversion due to different tools to evaluate it in the studies included in this meta-analysis and this can be considered another study limitation. We also could not assess the materials of stent as this was not reported in the studies included in our review.

## CONCLUSIONS

While both forms of urinary diversion can be utilized in malignant obstruction, our meta-analysis favours stents as the preferred choice as these are easier to maintain, and ureteral stent placement should be recommended whenever feasible. If the malignant obstruction precludes a stent placement, then percutaneous nephrostomy tube is a safe alternative. The findings of our review can help clinicians in using a personalized approach to choose either option in malignant ureteral obstruction.
